# Supplementary material for: Reliable crystal structure predictions from first principles
Source: Nat Commun. 2022 Jun 2;13:3095. doi: 10.1038/s41467-022-30692-y (PMC9163189; doi:10.1038/s41467-022-30692-y)
Supplement: Supplementary file 1 — Supplemental Information [file 41467_2022_30692_MOESM1_ESM.pdf]

Supplementary Information:
Reliable crystal structure predictions from first principles

Rahul Nikhar et al.

(Dated: May 9, 2022)

SUPPLEMENTARY TABLES AND FIGURES

The monomers used to validate the CSP(aiFF) protocol are shown in Supplementary Figure 1. The extended version of Table I from the main text is presented here as Supplementary Table 1, whereas Supplementary Table 2 compares the performance of the aiFF and the W99+charges FF. Supplementary Table 3 presents information about PESs developed in this work. Supplementary Figure 2 shows percentage errors of the cell parameters. The lattice energy vs. density landscapes for systems IV and XXII are displayed in Supplementary Figure 3. The ternary graph for attractive energies of all systems is shown in Supplementary Figure 4. The radial PES curves through the global minimum for systems II, IV, XVI, and nitromethane are shown in Supplementary Figure 5.

SUPPLEMENTARY DISCUSSION

Accuracy of aiFF versus pDFT+D. In the main text, we discuss the reasons why pDFT+D calculations improve the rankings of polymorphs relative to the rankings from the lattice energy minimization step with aiFFs. Two possible reasons mentioned there will be elaborated upon here. One reason could be that our SAPT(DFT) calculations were performed in modest-size basis sets, whereas the pDFT ones were performed with relatively high cutoffs of plane-wave basis sets and are better converged in basis-set size (an increase of the plane-wave cutoff from 340 to 612 eV, which significantly increases the completeness of this basis set, changed the lattice energy of system I by only 1%). To investigate this effect, we have computed SAPT(DFT) interaction energies for the minima 1 and 4 of system I with extrapolations to the complete basis set limit from the aug-cc-pVDZ and aug-cc-pVTZ plus midbond basis sets. The magnitude of interaction energy increased in both cases by 13%, but the ratio of depths of minimum 4 to 1, which is most relevant for CSPs, changed from 0.884 to 0.886, i.e., negligibly.

Another possible reason is that SAPT(DFT)-based aiFFs neglect pairwise nonadditive many-body effects, while such effects are partly included in pDFT+D calculations. However, only the induction (polarization) many-body effects are described reliably by DFT [1, 2]. To find out if there is a correlation between rankings and the expected magnitude of induction many-body effects, we have examined the physical components of interaction energies. Supplementary Figure 4 shows relative contributions of the (two-body) electrostatic, induction, and dispersion energies to the sum of attractive interactions at selected points on the PESs. Many-body induction effects can be substantial only for systems with large two-body induction effects. For the water dimer, the model systems with exceptionally large induction effects, the electrostatic, induction, and dispersion relative contributions at the van der Waals minimum are 0.62, 0.18, and 0.20 (Ref. 3). Supplementary Figure 4 shows that there are two systems similar to water in terms of these ratios: fluorouracil and system VIII, with aiFF-minimization ranks of 9 and 4, respectively. However, for fluorouracil we know that

the many-body effects were not the reason since the alt-CSP(aiFF) protocol, still two-body only, placed the experimental polymorph of this system at rank 1. While for system VIII the inclusion of induction many-body effects in pDFT+D may contribute to an improvement of rankings, the overall correlation between the magnitude of induction and rankings is poor, in particular, DNBT with rank 1 has a relatively large relative induction contribution, while system XIII with rank 4 is close to benzene, a prototypical dispersion-dominated system. Thus, it does not appear that the overall pDFT+D improvement of rankings is due to many-body effects. One may repeat here that it is possible to include the induction many-body effects in our approach using the polarization model and autoPES is capable of generating polarizable aiFFs.

Performance of an empirical FF. To compare the performance of the aiFF-based CSP approach with an approach using instead of aiFF an empirical FF, we chose the 2001 revision [4] of the W99 [5] model. W99 FF uses the standard Buckingham exp-6 potential: $A\mathrm{e}^{-\beta r} - C_6/r^6$, where r is an atom-atom distance and A, β, C_6 are empirical parameters, plus Coulomb interactions between point charges. The original W99 FF includes parametrized universal point charges, but later implementations of it, such a for example that of Ref. 6, used charges fitted to results of *ab initio* calculations for a specific monomer. We have followed this approach and used point charges from the "charges from electrostatic potentials on a grid" (CHELPG) method [7]. We will refer to this model as "W99+charges FF". The polymorphs already minimized at the OPLS FF stage of the CSP(aiFF) protocol were used as input to fine lattice energy minimizations with the W99+charges FF. In other words, the stage of the aiFF minimizations in UPACK was replaced by the stage with W99+charges FF minimizations (see Methods for the description of CSPs performed using UPACK). A detailed comparison of aiFF versus W99+charges FF performance is shown in Supplementary Table 1 and summarized in Supplementary Table 2. The latter table demonstrates that the performance of aiFFs is significantly better. For example, aiFFs have 94% of cases ranked at positions 10 or better, while W99+charges FF only 33%. This is indeed a qualitative differences from the point of view of technological applications. For two molecules, system XIII and fluorouracil, CSPs with W99+charges could not be done since the atom types needed (fluorine, bromine, and chlorine) are not available in this FF. CSPs based on W99+charges missed 3 out of 16 remaining polymorphs completely i.e., they failed to find the experimental crystal for system I (first polymorph), for system II, and for deferiprone (second polymorph) on the lowest lattice energy lists consisting of 3112, 568, and 2463, polymorphs, respectively. Clearly, some functional groups in these molecules are poorly represented by the W99+charges FF. One may also add that molecules like DNBT and TNB are not well parametrized in most empirical force fields. The W99+charges FF actually performs for these crystals better than other empirical FFs since the training set of W99 included a large number of nitrogen-containing molecules. Still, the RMSD_{20} is 0.81 Å for DNBT, slightly beyond the CCDC cutoff. W99+charges performs reasonably well for cell geometries. For the experimental crystal structures found on the W99+charges CSP lists,

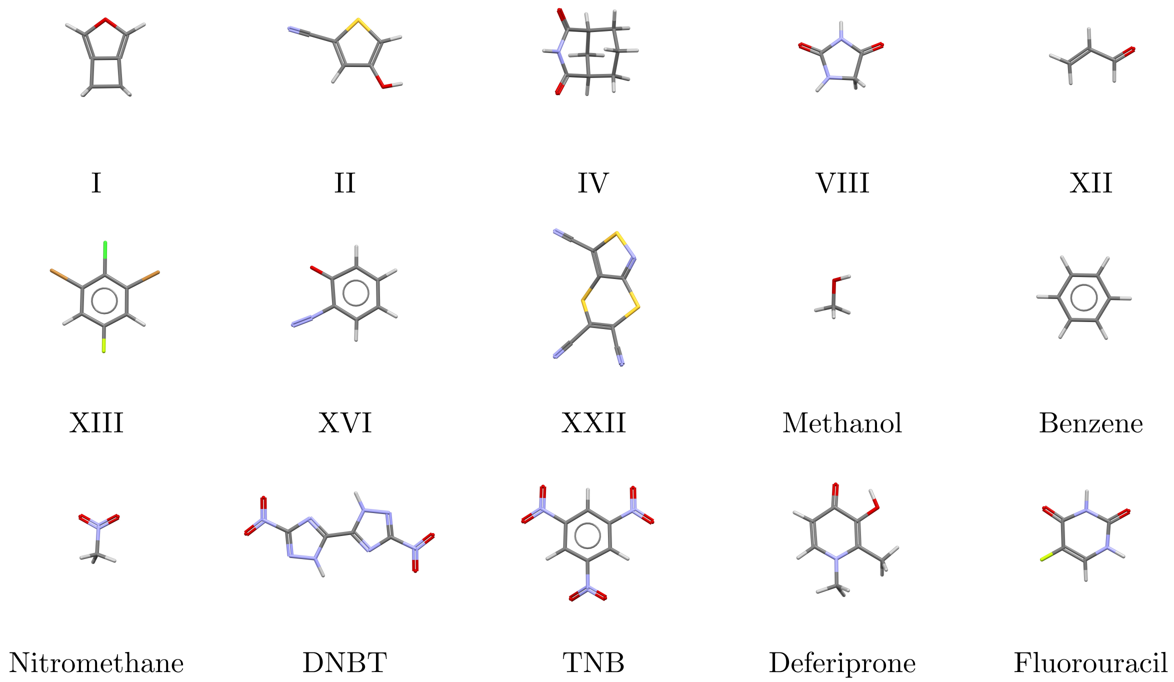

Supplementary Figure 1: **Molecular structures.** Graphs of the 15 investigated molecules. The roman numerals denote consecutive systems from the CCDC blind tests.

13 out of 18 cases, the average errors for the cell parameters a, b, c, and β amount to 5.6%, 3.3%, 5.5%, and 2.2%, respectively. This should be compared to the aiFF errors for the same set of structures: 4.0%, 2.9%, 5.1%, and 1.2%.

Supplementary Table 1: **CSPs from SAPT(DFT)-based aiFFs minimizations followed by pDFT+D fixed-geometry calculations and from W99+charges empirical FF minimizations.** In the 'System' column: 'Exp' denotes the experimental crystal structure and 'Calc' denotes the predicted crystal structure using aiFF or W99+charges FF (abbreviated as W99); SG: space group of the crystal; Rank for aiFF row: ranks of the experimental polymorph after aiFF minimizations and after pDFT+D calculations; Rank for W99 row: rank after W99+charges minimizations; $RMSD_{20}$: root mean square deviation (in Å) between the experimental crystal and the calculated polymorph for 20 overlapping molecules (heavy atoms only); RMSE: root mean square error (in kJ/mol) of the aiFF fit for negative interaction energies; ρ: density (in g/cm^3); a, b, c (in Å), α, β, γ (in degrees): cell parameters, α and γ are equal 90° for all systems; NF: experimental crystal was not found in the list of 500 or more (see text) lowest-energy structures; APU: some atom parameters unavailable in W99 force field; MTH, BZ, NM, DF, and FU: abbreviations for methanol, benzene, nitromethane, deferiprone, and fluorouracil, respectively.

System	SG	FF	Rank	$RMSD_{20}$	RMSE	ρ	a	b	c	β
$I_{Exp-Poly1}$	$P2_1/c$					1.324	4.954(2)	9.845(2)	9.679(2)	90.57(4)
I_{Calc}	$P2_1/c$	aiFF	2/1	0.09	0.6	1.328	4.992	9.895	9.531	89.37
		W99	NF	-	-					
$I_{Exp-Poly2}$	$Pbca$					1.280	5.309(10)	12.648(2)	14.544(3)	90.00
I_{Calc}	$Pbca$	aiFF	8/2	0.32	0.6	1.337	5.216	12.546	14.293	90.00
		W99	1	0.27	-	1.193	5.387	12.897	15.078	90.00
II_{Exp}	$P2_1/n$					1.489	7.516(2)	8.332(2)	9.059(2)	100.19(3)
II_{Calc}	$P2_1/n$	aiFF	1/1	0.59	1.3	1.575	7.154	8.402	9.041	103.80
		W99	NF	-	-					
IV_{Exp}	$P2_1/a$					1.338	7.7046(5)	10.6062(7)	9.3384(6)	95.033(2)
IV_{Calc}	$P2_1/c$	aiFF	2/1	0.24	0.63	1.363	8.970	10.597	7.896	96.16
		W99	17	0.27	-	1.274	9.148	10.784	8.128	95.25
VIII_{Exp}	$C2/c$					1.669	9.3538(7)	12.1757(11)	7.2286(6)	104.593(4)
VIII_{Calc}	$C2/c$	aiFF	4/1	0.28	1.1	1.669	8.981	12.085	7.4389	99.50
		W99	7	0.54	-	1.505	9.056	12.107	8.152	98.74
XII_{Exp}	$Pbca$					1.084	7.134(8)	9.694(11)	9.930(10)	90.00
XII_{Calc}	$Pbca$	aiFF	9/1	0.53	0.84	0.969	7.328	9.393	11.163	90.00
		W99	14	0.24	-	1.066	6.788	9.990	10.307	90.00
XIII_{Exp}	$P2_1/c$					2.528	3.8943(5)	13.5109(17)	14.4296(17)	93.636(2)
XIII_{Calc}	$P2_1/c$	aiFF	4/1	0.45	1.1	2.334	4.064	13.989	14.449	87.38
		W99	APU	-	-					

Continued on next page

Supplementary Table 1 – continued from previous page

System	SG	FF	Rank	$RMSD_{20}$	RMSE	ρ	a	b	c	β
XVI_{Exp}	*Pbca*					1.385	9.6451(18)	7.3810(14)	16.185(3)	90.00
XVI_{Calc}	*Pbca*	aiFF	16/1	0.29	1.0	1.395	9.21	7.547	16.46	90.00
		W99	24	0.21	-	1.295	9.910	7.458	16.672	90.00
XXII_{Exp}	$P2_1/n$					1.727	11.947(2)	6.696(10)	12.598(3)	108.60(3)
XXII_{Calc}	$P2_1/n$	aiFF	1/1	0.15	1.4	1.748	11.959	6.713	12.414	108.79
		W99	39	0.48	-	1.660	11.525	6.400	12.327	107.210
MTH_{Exp}	$P2_12_12_1$					1.028	4.6469(9)	4.9285(10)	9.0403(18)	90.00
MTH_{Calc}	$P2_12_12_1$	aiFF	6/1	0.40	0.92	1.045	5.074	4.783	8.393	90.00
		W99	3	0.38	-	0.905	5.293	5.009	8.865	90.00
$BZ_{Exp-Poly1}$	*Pbca*					1.070	7.3801(15)	9.5154(19)	6.9029(14)	90.00
BZ_{Calc}	*Pbca*	aiFF	1/1	0.16	0.59	1.015	7.483	9.486	7.202	90.00
		W99	31	0.27	-	1.029	7.063	9.540	7.551	90.00
$BZ_{Exp-Poly2}$	$P2_1/c$					1.195	5.5146(11)	5.4951(11)	7.6536(15)	110.59(3)
BZ_{Calc}	$P2_1/c$	aiFF	4/3	0.40	0.59	1.036	5.688	5.670	8.373	111.95
		W99	4	0.39	-	1.052	5.598	5.686	8.078	106.506
NM_{Exp}	$P2_12_12_1$					1.545	5.18579(3)	6.23660(4)	8.51273(6)	90.00
NM_{Calc}	$P2_12_12_1$	aiFF	1/1	0.27	0.74	1.379	5.113	6.525	8.811	90.00
		W99	2	0.18	-	1.395	5.146	6.423	8.793	90.00
$DNBT_{Exp}$	$P2_1/n$					1.902	5.0559(6)	6.3080(7)	12.4268(14)	95.136(11)
$DNBT_{Calc}$	$P2_1/c$	aiFF	1/1	0.58	1.56	1.882	4.874	6.785	12.120	95.48
		W99	30	0.81	-	1.909	4.663	7.159	11.907	98.199
TNB_{Exp}	$P2_1/c$					1.717	12.896(5)	5.723(2)	11.287(5)	98.190(8)
TNB_{Calc}	$P2_1/c$	aiFF	3/1	0.67	1.28	1.621	12.745	6.213	11.472	105.95
		W99	100	0.47	-	1.678	13.239	6.051	10.589	83.89
$DF_{Exp-Poly1}$	*Pbca*					1.429	7.253(2)	12.989(4)	13.736(4)	90.00
DF_{Calc}	*Pbca*	aiFF	2/2	0.28	0.71	1.412	7.398	12.559	14.093	90.00
		W99	2	0.34	-	1.333	7.750	12.636	14.158	90.00
$DF_{Exp-Poly2}$	$P2_1/c$					1.465	6.4847(10)	13.6853(3)	7.1462(10)	95.6583(8)
DF_{Calc}	$P2_1/c$	aiFF	8/1	0.24	0.71	1.410	6.408	14.082	7.273	87.044
		W99	NF	-	-					
FU_{Exp}	$P2_1/c$					1.791	5.154(16)	15.001(5)	6.654(2)	110.336(5)
FU_{Calc}	$P2_1/c$	aiFF	9/1	0.61	1.06	1.727	5.939	15.278	6.210	117.391
		W99	APU	-	-					

Supplementary Table 2: Comparison of ranking of the 18 polymorphs by aiFF and by W99+charges FF. The numbers give the percent of the polymorphs at a given range of ranks, with cumulative values in parentheses. APU denotes missing parameters in W99 FF. The column >100 counts CSPs that did not include the experimental polymorph within 3112, 568, and 2463 lowest lattice energy polymorphs for monomers I, II, and deferiprone, respectively.

	1	2–10	11–20	21–100	>100	APU
aiFF	28	67 (94)	6 (100)			
W99+charges	6	28 (33)	11 (44)	28 (72)	17 (89)	11 (100)

Supplementary Table 3: RMSEs (in kJ/mol) of PESs evaluated on subsets of the close-range grid points. Numbers of grid points in the subsets are given in parentheses. The number of free parameters in the close-range fitting stage is denoted N_{FP}, the number of detected minima of the PES by N_{min}, and the total number of grid points by N_{grid}.

System	N_{grid}	N_{FP}	$N_{\mathrm{grid}}/N_{\mathrm{FP}}$	N_{min}	RMSE $E_{\mathrm{int}} < 0$
I	642	70	9.2	17	0.6 (396)
II	872	88	9.9	10	1.3 (559)
IV	1693	270	6.3	126	0.63 (915)
VIII	2982	154	19.4	36	1.1 (1322)
XII	507	88	5.7	7	0.84 (248)
XIII	564	88	6.4	35	1.1 (333)
XVI	2792	208	13.4	9	1.0 (1859)
XXII	2413	330	7.3	80	1.4 (1673)
Methanol	1081	54	20	3	0.92 (703)
Benzene	391	10	39	3	0.59 (188)
Nitromethane	593	70	8.5	10	0.74 (268)
DNBT	5155	378	13.6	43	1.56 (3207)
TNB	781	25	31.2	235	1.28 (524)
Deferiprone	6113	418	14.6	13	0.71 (4120)
Flurouracil	1828	180	10.2	16	1.06 (1023)

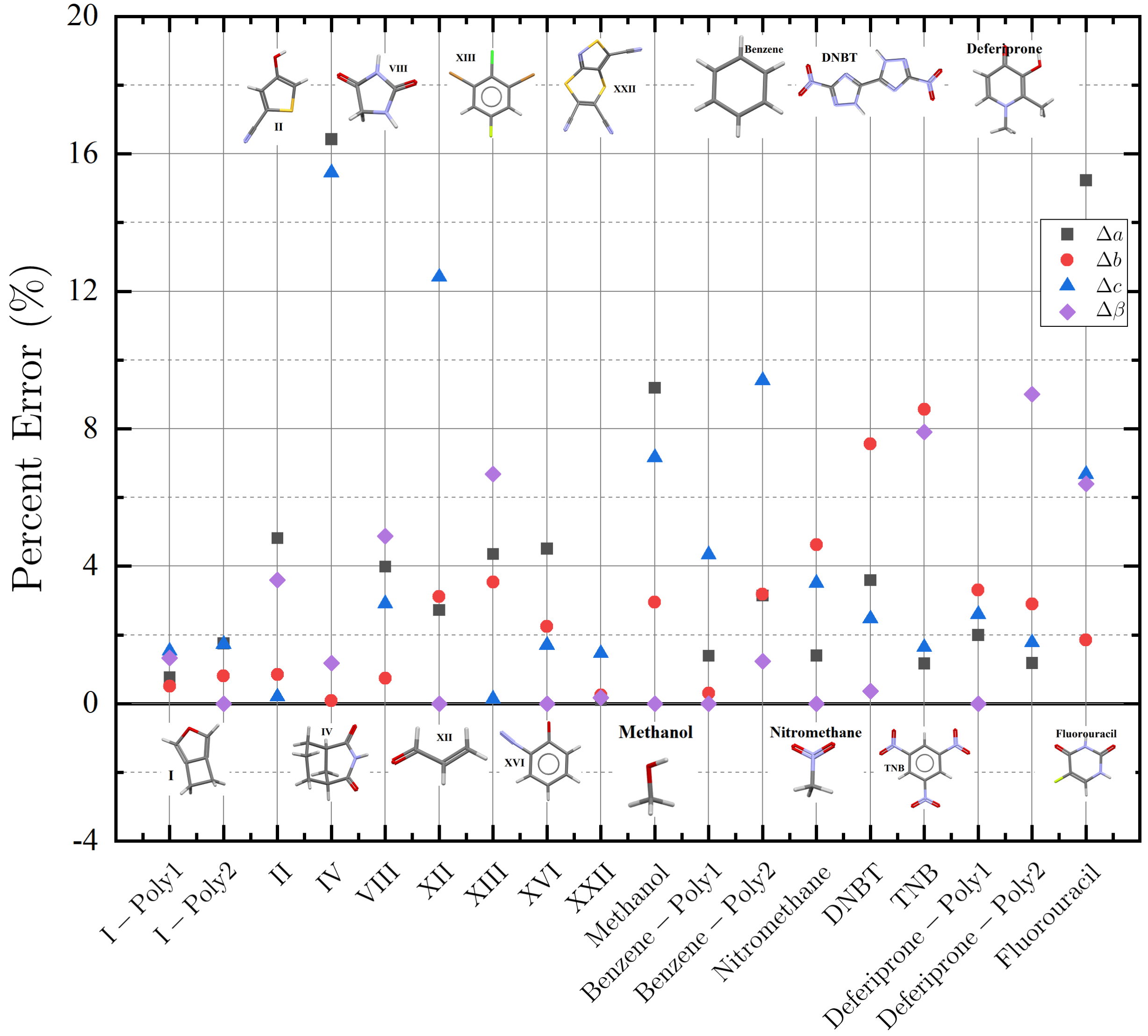

Supplementary Figure 2: **Accuracy of cell parameters.** Percentage errors for 18 polymorphs that match the experimental ones.

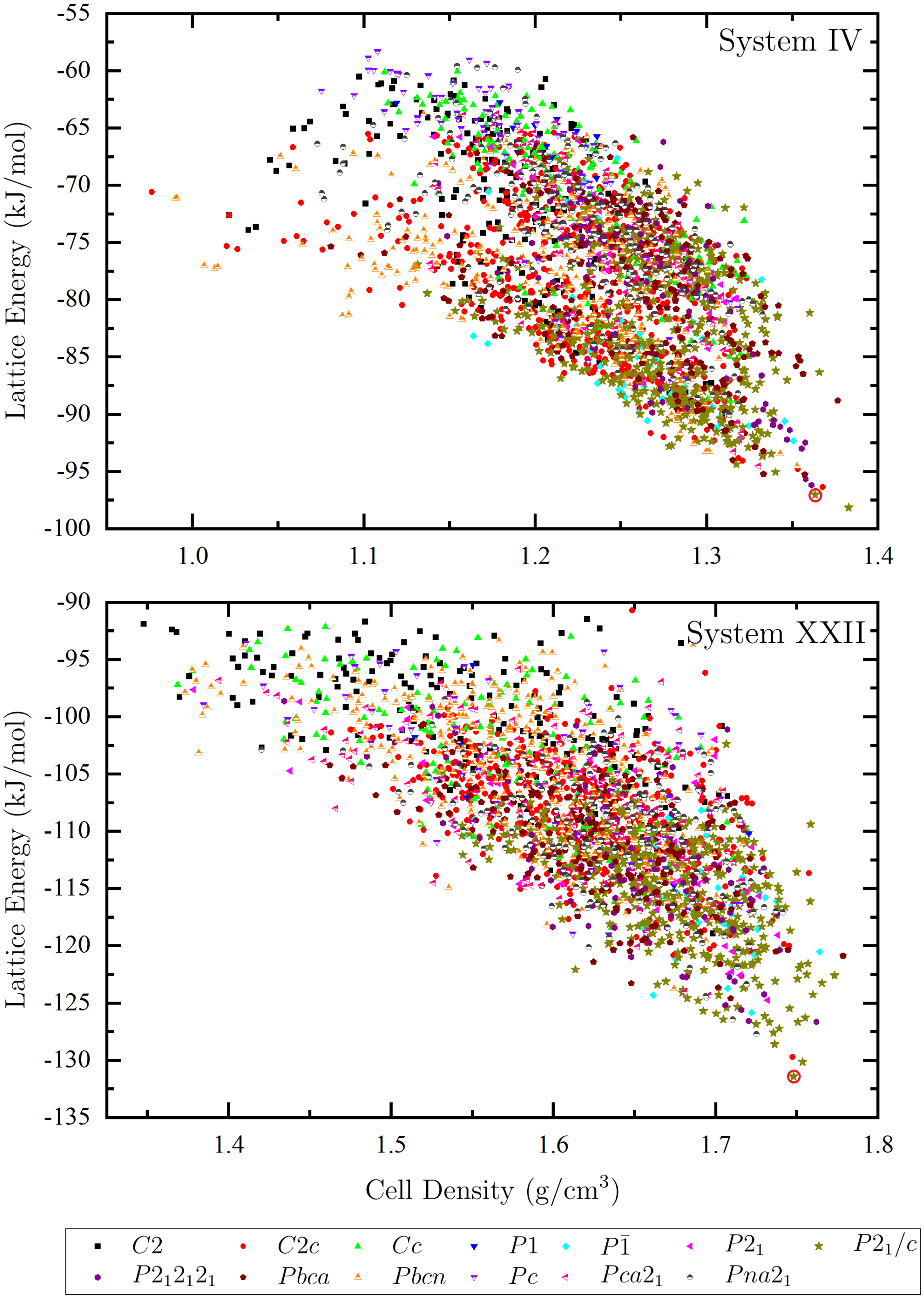

Supplementary Figure 3: **Lattice energy vs. density.** Polymorph lattice energy vs. density landscape for systems IV and XXII from SAPT(DFT)-based aiFF lattice energy minimizations. The polymorphs closest to the experimental crystal structures are marked by red circles.

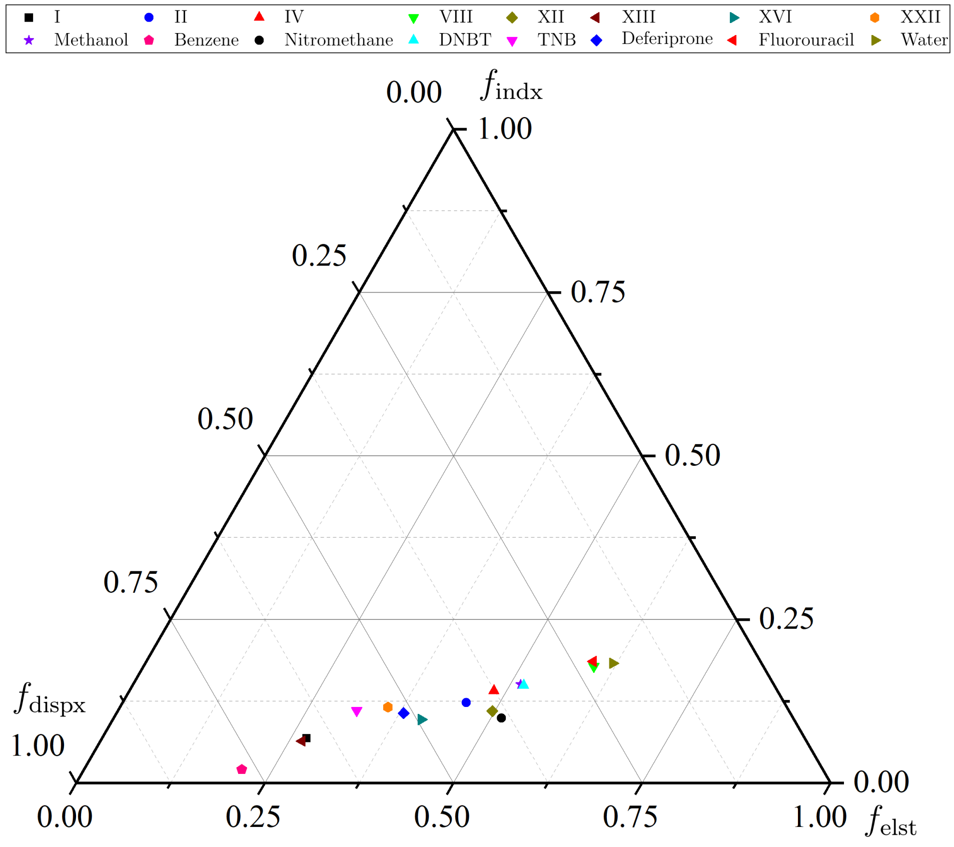

Supplementary Figure 4: **Decomposition of interaction energies.** The vertices of the ternary graph represents f_{indx}, f_{dispx}, and f_{elst}, where f_{X} is the ratio of the interaction energy component X to the sum of the three components. See Methods for definitions of components. Each point represents the ratio of the average of these components to the average of their sum for the following five points on the PESs: the three lowest minima and the nearest and second nearest neighbour dimer configurations from the polymorph that matches best the experimental crystal.

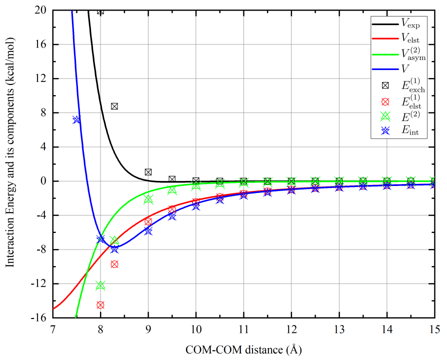

II

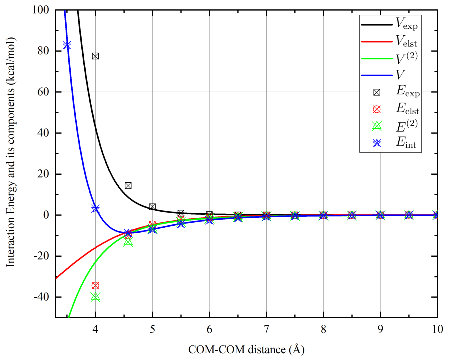

IV

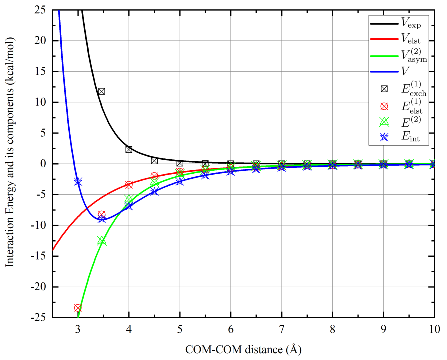

XVI

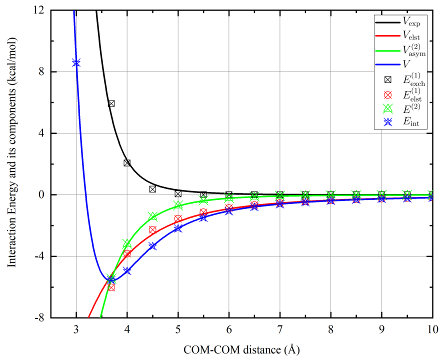

Nitromethane

Supplementary Figure 5: **Radial graphs.** Radial dependence of SAPT(DFT) interaction energy and its components for the orientation corresponding to the global minimum of systems II, IV, XVI, and nitromethane. The solid lines and symbols represents PES and SAPT(DFT) energies, respectively. Energy components $E^{(1)}_{\text{exch}}$, $E^{(1)}_{\text{elst}}$, and $E^{(2)}$ represent the first-order exchange component, the electrostatic component, and the sum of "indx" and "dispx" components, respectively. Correspondingly, V_{exp}, V_{elst}, and $V^{(2)}_{\text{asym}}$ represent the fit components corresponding to the exchange, electrostatic, and the sum of induction and dispersion energies, respectively. $E^{(2)}$ and $V^{(2)}_{\text{asym}}$ include contributions from $\delta E^{\text{HF}}_{\text{int,resp}}$.

Supplementary References

[1] Hapka, M. *et al.* The nature of three-body interactions in DFT: Exchange and polarization effects. *J. Chem. Phys.* **147**, 084106 (2017).

[2] Jankiewicz, W., Podeszwa, R. & Witek, H. A. Dispersion-corrected DFT struggles with predicting three-body interaction energie. *J. Chem. Theory Comput.* **14**, 5079–5089 (2018).

[3] Patkowski, K., Szalewicz, K. & Jeziorski, B. Third-order interactions in symmetry-adapted perturbation theory. *J. Chem. Phys.* **125**, 154107–(1:20) (2006).

[4] Williams, D. E. Improved intermolecular force field for molecules containing H, C, N, and O atoms, with application to nucleoside and peptide crystals. *J. Comput. Chem.* **22**, 1154–1166 (2001).

[5] Williams, D. E. Improved intermolecular force field for crystalline hydrocarbons containing four- or three-coordinated carbon. *J. Mol. Struct.* **485–486**, 321–347 (1999).

[6] Day, G. M., Motherwell, W. D. S. & Jones, W. Beyond the isotropic atom model in crystal structure prediction of rigid molecules: Atomic multipoles versus point charges. *Cryst. Growth Des.* **5**, 1023–1033 (2005).

[7] Breneman, C. M. & Wiberg, K. B. Determining atom-centered monopoles from molecular electrostatic potentials. The need for high sampling density in formamide conformational analysis. *J. Comp. Chem.* **11**, 361 (1990).
